# Potential effects of ursodeoxycholic acid on accelerating cutaneous wound healing

**DOI:** 10.1371/journal.pone.0226748

**Published:** 2019-12-23

**Authors:** Tarek El-Hamoly, Sahar S. Abd El-Rahman, Megahed Al-Abyad

**Affiliations:** 1 Drug Radiation Research Department, National Center for Radiation Research and Technology, Atomic Energy Authority, Cairo, Egypt; 2 Cyclotron Project, Nuclear Research Centre, Atomic Energy Authority, Cairo, Egypt; 3 Department of Pathology, Faculty of Veterinary Medicine, Cairo University, Giza, Egypt; NCMLS, Radboud University Nijmegen Medical Center, NETHERLANDS

## Abstract

Among the initial responses to skin injury, triggering inflammatory mediators and modifying oxidative status provide the necessary temple for the subsequent output of a new functional barrier, fibroplasia and collagen deposition, modulated by NF*-*κB and TGF-β1 expressions. Hence, the current study aimed to investigate the effect of local application of ursodeoxycholic acid (UDCA) on cutaneous wound healing induced in Swiss mice. Wound contraction progression was monitored by daily photographing the wounds. Enhanced fibroblast cell migration was observed after incubation with UDCA. Topical application of UDCA (500 μM) cream on excised wounds significantly enhanced wound contraction and improved morphometric scores. In addition, UDCA ameliorated the unbalanced oxidative status of granulated skin tissues. Interestingly, it showed increased expression of TGF-β1 and MMP-2 with decreased expression of NF*-*κB. On the other hand, UDCA significantly increased collagen fibers deposition and hydroxyproline content and enhanced re-epithelization. UDCA also modified the mitochondrial function throughout the healing process, marked by lower consumption rates of mitochondrial ATP, complex I contents as well as intracellular NAD^+^ contents accompanied by elevated levels of nicotinamide compared to the untreated controls. In chronic gamma-irradiated (6Gy) model, the illustrated data showed enhanced wound contraction via increased TGF-β1/MMP-2 and collagen deposition incurred by topical application of UDCA without effect on NF*-*κB level. In sum, the present findings suggest that UDCA may accelerate wound healing by regulating TGF-β1 and MMP-2 and fibroplasia/collagen deposition in either the two wound healing models.

## Introduction

Although the molecular mechanisms underlying the pathology of wound healing are extensively considered in many studies, the urge for new therapeutic targets is of utmost importance for the successful treatment of wound healing abnormalities and avoidance of the consequences of wound healing complications. The formally recognized cutaneous wound healing phases emphasize the aetiology of inflammatory responses, molecular factors recruitment and cellular event development that start with re-epithelialization, fibroplasia and angiogenesis and end with scar formation. A pivotal role is well known to be played by Nuclear factor κB (NF-κB) during the expression of various inflammatory cytokines, inflammatory mediators, adhesion molecules and chemokines **[1].** During the remodelling phase, a new extracellular matrix (ECM) is synthesized that is characterized by collagen and elastin fibers accumulation, an action that principally depends upon the relative expression of fibrogenic (TGF-β1) to fibrolytic matrix metalloproteinases (MMPs) and their tissue inhibitors recruitment to build up the wound scar **[2].** Additionally, targeting, mobilization, and particularly extracellular activation of the transforming growth factor-β1 (TGF-β1) family are the dominant regulators for other biological actions involved in the healing process. On the other hand, exposure to gamma radiation was found to interfere with normal wound healing. Such observation was previously explained by mechanisms of inflammatory markers overexpression, extreme ROS production, fibroplasia, and variation in the levels of regulatory growth factors **[3].**

3α, 7β-dihydroxy-5β-cholanicacid (UDCA) is a hydrophilic dihydroxy bile acid that is commercially available for various hepato-cholestatic disorders remediation **[4].** Previous studies have examined the non-hepatic effects of UDCA on multiple pathophysiological models such as eosinophilic airway inflammation **[5]**, colorectal carcinoma **[6]**, parkinsonism **[7]**, etc. highlighting the advantageous and versatile influences relating to anti-inflammatory effects **[8]**, antioxidant effects **[9]**, and counteracting the propagation of apoptotic pathways as well as mitochondrial biogenesis **[10, 11].** Moreover, UDCA is recognized as NF-κB suppressor particularly through the functional modification of the glucocorticoid receptor and its dependent transcription on NF-κB **[12].** The present study aimed to investigate the possible signalling pathways through which UDCA could influence the process of wound healing.

## Materials and methods

### Preparation of mouse embryonic fibroblast (MEF) cell separation and scratch assays

All chemicals used were obtained from Sigma-Aldrich (St. Louis, MO, USA). Embryos from Balb/c mice were obtained on day thirteen of gestation according to the method previously described by Wang et al. **[13].** Briefly, the pregnant females were sacrificed through euthanasia using an overdose of isoflurane. The animals were placed on their backs, and the abdomens were swabbed with 70% ethanol for further midline laparotomy. After locating the reproductive tract, the uteri-containing embryos were surgically dissected. Afterward embryos were placed in a centrifuge tube after removal of all vital organs and mincing with scissors. Five millilitres of 0.25% trypsin were added to the tube and incubated at 37°C for 20 min. The trypsin was inactivated using 5 ml of Dulbecco's modified Eagle medium (DMEM) supplemented with 10% foetal bovine serum. The cells of the embryos were centrifuged (1,500 xg for 5 min at room temperature) and suspended in 15 ml of a fresh medium. After standing for 10 min, the top layer of cell suspension was collected and plated in a 100-mm dish to be cultured at 37°C in a humidified atmosphere containing 5% CO_2_.

Confluent cultures of mouse fibroblasts seeded in 96-well plates were scratched with a small pipette tip. Damaged cells were removed by washing with PBS solution. A triplicate of freshly wounded samples was dried and used as a control (0% healed samples). The data analysis was carried out based on two controls; initial (T = 0) control which was evaluated by microscopic imaging directly after scratching to compare the original unoccupied area among treated and untreated wounded cells. The second one was the untreated control (T = 24) recorded for cell migration after 24 h and used to compare all treated groups. Cells were treated with glucose (20 mM), 1,5-isoquinolinediol (DIQ, 100 μM) **[13, 14]** or UDCA (50 μM) **[15]** and incubated for 24 h. Samples were then washed twice with PBS, and images were acquired under a Zeiss AxioVision microscope (Carl Zeiss Microscopy GmbH, Jena, Germany). Images were analysed with Tscratch software. The ratio of the covered area was compared with that of freshly wounded samples.

### Animal experiments

#### Animals

Male Swiss mice (6–8 weeks old) were housed individually under 12 h light/dark cycle standard conditions Animal experiments were conducted according to the protocol approved by the Institutional Animal Care and Use Committee at Cairo University (IACUC, CU II F65 18).

#### Wound incision and treatment protocol

The wound incision procedure was performed as previously described by El-Hamoly et al. **[16].** Briefly, the back skin of each animal was shaved and cleaned with 70% alcohol. Mice were kept warm during isoflurane (Abbott Animal Health, Abbott Park, IL, USA) inhalation anaesthesia and surgery using a heat lamp. Two 6-mm full-thickness, wounds were incised on the back of the animal and swabbed with povidone-iodine to inhibit further contamination. A single s.c. dose of ketoprofen (5 mg/ml) was given to alleviate post-surgical pain. UDCA cream (500μM/ day) (SEDICO Co., Cairo, Egypt) was applied daily to one of the wound circles. Dose selection was inferred by a dose-dependent experiment on wound contraction assay (S1 Fig). The UDCA cream was prepared by dissolving in 10% DMSO to make 500 mM solution. The prepared solution was then incorporated in hydrophilic cream (El Gomhouria Co. For Drugs & Medical Supplies, Cairo, Egypt) till homogeneous and uniform appearance obtained (2% cream). The other wound circle was treated with the vehicle and served as a negative control.

#### Estimation of wound contraction

Daily photographs of the two excised wounds (*n = 6*) were taken starting on day zero and recoded for successive 10 days. Using NIH ImageJ software (ImageJ, 1.46a, NIH, USA), the images were analysed to calculate the ratio of wound area closure to the original wound area on the day of wounding (day zero) for either treated or untreated wounds.

#### Tissue harvesting

On day zero and day 5 post-wounding, mice (n = 6 per time point) were euthanasia sacrificed via an overdose of isoflurane inhalation then confirmed by cervical dislocation. Tissue specimens from the surrounding area of the wound circle were collected at the assigned time points; one portion was stored in 10% neutral buffered formalin for histopathological studies, and the other portion was snap-frozen in liquid nitrogen for further biochemical analysis. Protein contents were quantified according to BCA protein assay protocol (Pierce, Rockford, Illinois, USA).

### Biochemical analysis

#### Determination of hydroxyproline

The hydroxyproline concentration was determined as previously mentioned by Woessner **[17].** For estimation of total collagen content, skin tissue samples were hydrolysed in 6N HCl for 3 h at 130°C, neutralized to pH 7 with 2.5N NaOH and diluted with Milli-Q water. Afterward, the solution was mixed with chloramine-T reagent and incubated for 20 min at room temperature. Thereafter, freshly prepared r-dimethylamino-benzaldehyde (Ehrlich’s reagent) solution was added and incubated for further 15 min at 60°C. The absorbance of samples was measured at 550 nm. The amount of hydroxyproline was determined by plotting a standard curve. The collagen content of the skin tissues was expressed as μg/mg total protein.

#### Oxidative stress evaluation

Hydrogen peroxide (H_2_O_2_) elimination was measured in skin wound tissue homogenates as an indicator of oxidative stress using a colorimetric kit (Bio-diagnostic Co., Cairo, Egypt). Briefly, samples were homogenized in potassium phosphate buffer solution (pH 7.5, 1mM EDTA). The skin mixture was minced with a homogenizer (10,000 rpm/ 5 min). Afterward, the sample was incubated at 37°C for 2 hours and then centrifugation at 3,000 rpm/ 3 min. Acetonitrile was added to the supernatant to deproteinize the sample and then centrifuged again. After this, a 100 μL of the supernatant was taken and mixed with 900 μl of the reaction solution containing 250 μM ammonium ferrous sulphate, 100 μM xylenol orange and 100 μM sorbitol in 25 mM H_2_SO_4_. Then it was kept at room temperature for 30 min. The absorbance was detected at 510 nm by a UVD-2950 spectrophotometer. Catalase activity was also assayed according to the method of Luck **[18].** The changes in absorbance were recorded at 240 nm/min over 5 min and the activity was calculated as unit/mg protein.

#### Nucleotides estimation and mitochondrial function evaluation

The mitochondrial fraction was isolated according to Dimauro et al. **[19],** ATP extraction was performed by the addition of 1.75 mL of 60% perchloric acid (denaturation) to 100 μg of purified mitochondrial fractions and left on ice for 10 min then centrifuged (15300 xg for 5 min). Next, 60 μL of supernatant was neutralized with 11.5 μL of 1 M KOH and incubated on ice for 3 min; then, the reaction was centrifuged as before. ATP extracts were quantified using high-performance liquid chromatography (HPLC) according to previously described method **[20].** To determine the mitochondrial complex I activity, the reaction mixture was prepared by mixing 67.5ml of BSA, 0.5mL of 0.04 mMCo-Q1, 5.5mL 2mMof 2 mM NaN_3_, 1.5mL of 0.1Mm EDTA and 122mL of20 mM KH_2_PO_4_. Then, 50 μL of the mitochondrial fraction was mixed with 0.9ml of the reaction mixture and left at room temperature for 3 min to reach equilibrium. NADH (0.1mL) was added to initiate the reaction. The decrease in absorbance per min was monitored at 340 nm for 3 min.NAD^+^ and nicotinamide (NAM) contents were extracted and determined as previously described by Yoshino and Imai **[21].** Peaks were separated at λ = 254 nm and the concentrations were expressed as μM/mg protein content.

#### Histopathological studies

Formalin-fixed skin wounds and the surrounding areas were subjected to routine dehydration in graded concentration of alcohol, cleared in xylene, and finally embedded in paraffin. Paraffin blocks were cut into 5-μm thickness. The obtained sections were mounted on glass slides, routinely stained with H&E **[22]** and subjected to histopathological examination using an Olympus BH2 electric light microscope (Tokyo, Japan). The degree of re-epithelialization (for formation of new epithelial layers), the number of cells with obvious nuclei, and the number of hair follicles were all scored between 0 and 3 (where 0 denotes no change and 1, 2, and 3 denote mild, moderate and severe changes, respectively) **[23]** in five separate microscopic fields. Van Gieson’s stain was used to evaluate type 1 collagen fibers deposition, which was scored between 0 and 3. The area percent of red-stained collagen fibers by Van Gieson’s stain was quantified using image analysis software (ImageJ, 1.46a, NIH, USA) in 5 high power microscopic fields.

### Immunohistochemical evaluation of TGF-β1, NF-κB and MMP-2

For recognition of TGF-β1, NF-κB and MMP-2 expression, 4-μm paraffin sections of skin wound specimens of control and different treatment groups were subjected to immunohistochemical procedures using avidin-biotin peroxidase as described by Hsu et al.**[24].** Briefly, paraffin sections were deparaffinized in toluene, rehydrated in ethanol, and then incubated with H_2_O_2_ to block endogenous peroxidase activity. The sections were incubated with monoclonal antibodies for TGF-β1, NF-κB and MMP-2 (Dako Corp, CA, USA) at dilutions of; 1:150, 1:200 and 1:50 respectively as recommended by the manufacturer. Haematoxylin (Sigma-Aldrich) was used as a counterstain for nuclei. Chromogen 3,3-diaminobenzidine tetrahydrochloride (DAB, Sigma-Aldrich, MO, USA) was used for visualization of the reactive cells for each marker. The immunohistochemical quantification of TGF-β1, MMP-2 and NF-κB was carried out by image analysis software (ImageJ, 1.46a, NIH, USA) through measuring the optical density of each marker in five different high-power microscopic fields. The area for each microscopic field was 18.8913mm^2^.

### Gamma-irradiation of mice to induce a chronic wound model

Male Swiss mice (6–8 weeks old) were subjected to acute whole-body gamma radiation (*n = 8*) *at* dose levels of 6 Gy according to the protocol approved by the Animal Care Committee of the National Centre for Radiation Research and Technology (NCRRT), Egyptian Atomic Energy Authority, Egypt. Twenty-four hours later, incision of wounds on the irradiated animals was carried out and treated as previously described. Wound contraction analysis for daily acquired images and further histological studies were performed according to the mentioned protocols.

### Statistical analysis

**Fig 1:**
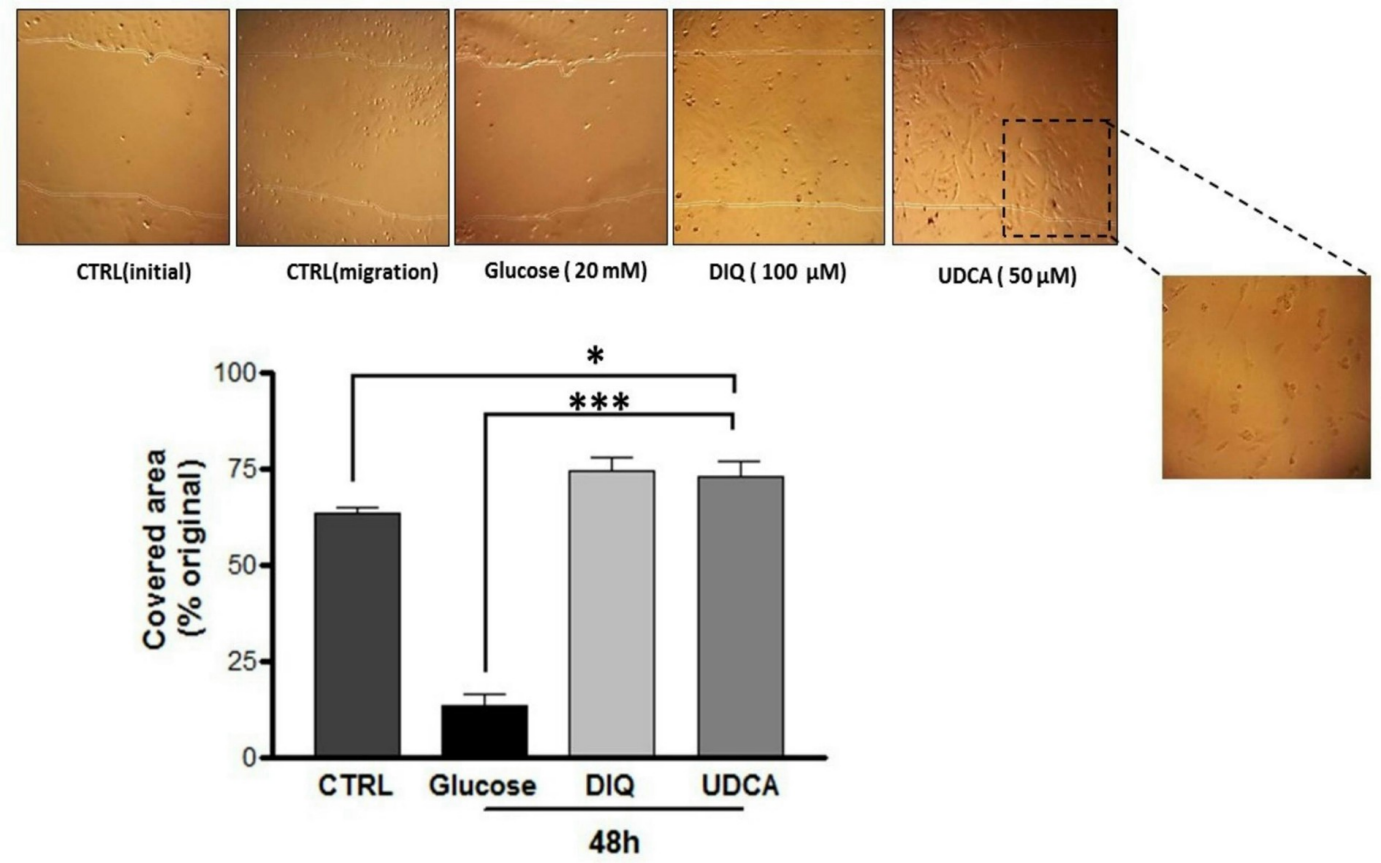
UDCA stimulated fibroblast migration. Confluent cultures of mouse embryonic fibroblast were scratched with a sterile pipette’s tip and then incubated with glucose (20 mM), DIQ (100μM) or UDCA (50μM). Untreated cells served as control. Forty-eight hours later, photographs were taken, and images were analyzed for cells repopulation toward the scratched areas. *P<0.*05*, ***P < 0.*001*: significantly different from untreated control or negative control (glucose). DIQ: 1,5-isoquinolinediol, UDCA: ursodeoxycholic acid.

**Fig 2:**
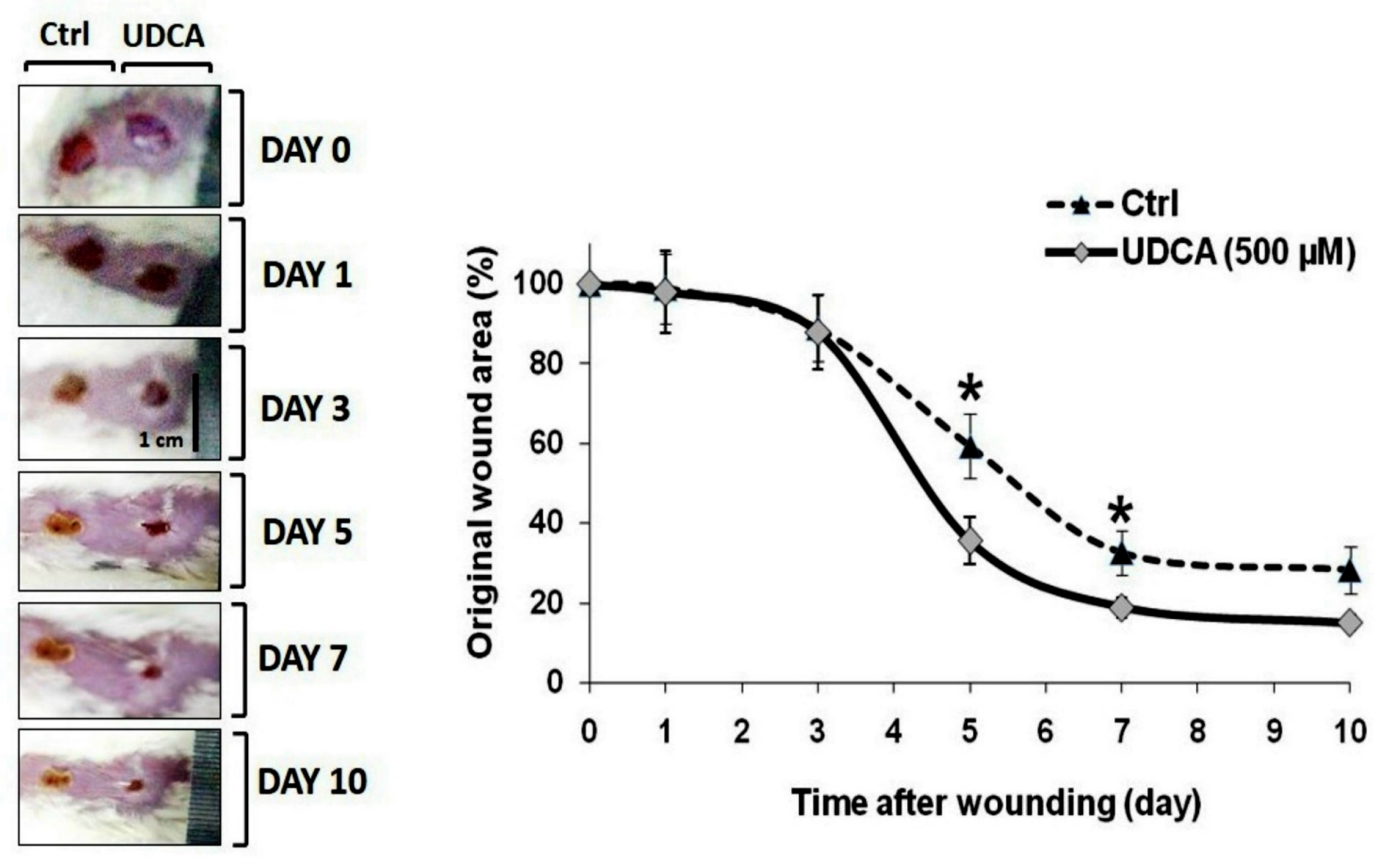
Effect of UDCA on wound healing contraction. Two full thickness cutaneous wounds (6mm in diameter) were cut in male Swiss mice. Photos of wounds were captured every day starting from day 0 (wounding). Animals were treated with either vehicle (left wound) or UDCA (500 μM cream, right wound) for 10 consecutive days. The line graph represents the mean of percent change relative to original wound size ± SE median (n = 6). *P<0.*05*: significantly different versus vehicle-treated (dashed line) wounds in the same time point. UDCA: ursodeoxycholic acid.

All data except others mentioned were compared via one-way analysis of variance (ANOVA), followed by the Tukey-Kramer test for multiple comparisons. The difference between two groups was determined using student *t*-test for parameters measured in Figs 1 and 2. The results were expressed as the means ± SEM and considered statistically significant at *P* value <0.05.

## Results

### UDCA stimulated fibroblast migration

Proliferation and migration of fibroblasts are important factors for granulation tissue formation. In the *in vitro* scratch assays, UDCA facilitated the migration of cultured fibroblast cells and thus the repopulation of the scratched surface. The later effect was comparable to that induced by the negative (glucose) and positive controls (PARP-1 inhibitor, DIQ) (Fig 1).

### UDCA application accelerated wound contraction

Wound contraction of UDCA-treated mice showed a faster contraction starting on the third day compared to the untreated control. On the 5^th^ day, topical UDCA application revealed about 65% wound contraction relative to the original wound area compared with the untreated control wounds which showed only 40% wound contraction (Fig 2).

### UDCA application enhanced wound healing

**Fig 3:**
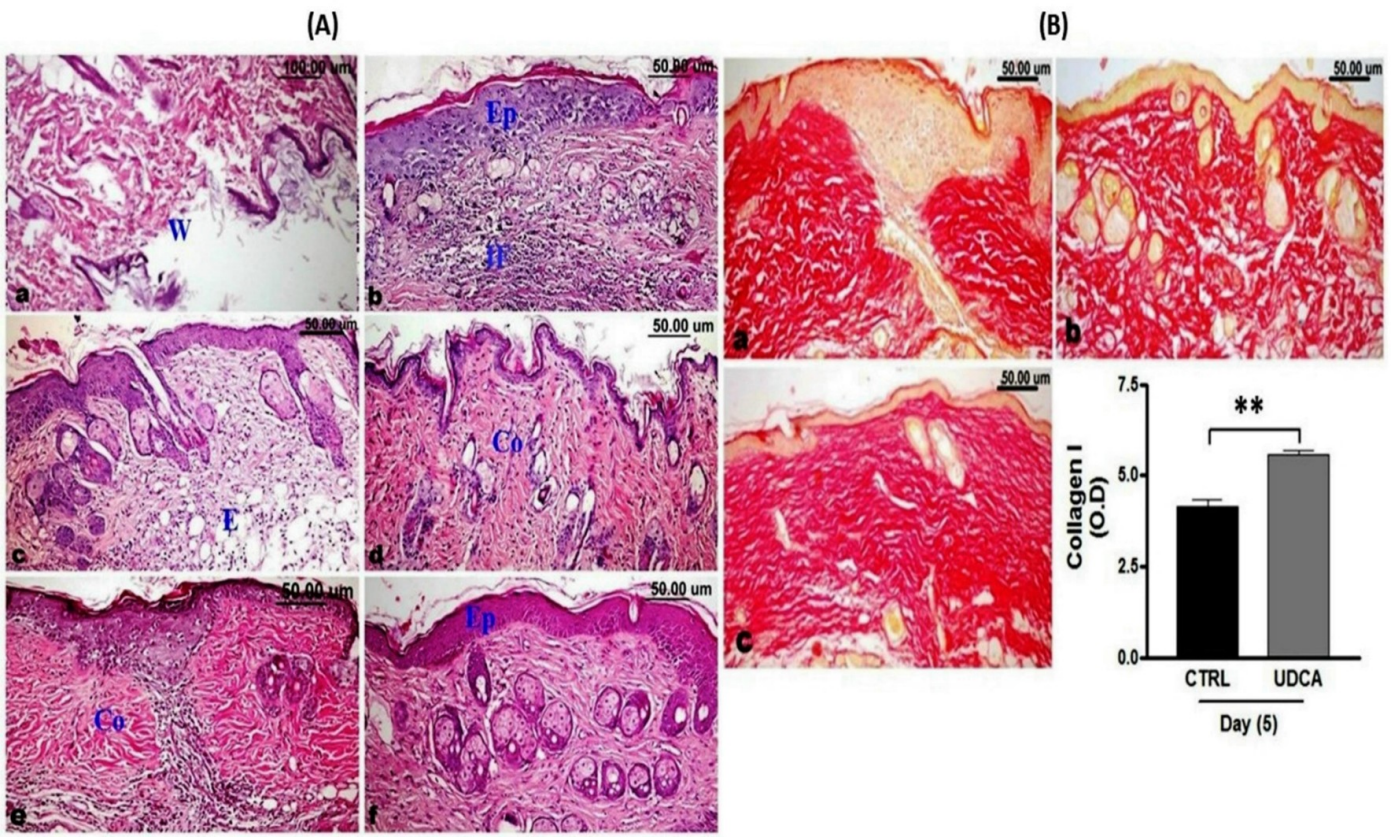
**(A).** Effect of UDCA on histopathological changes during wound healing (H&E stained sections). (a) At zero-day post wounding showing destructed epidermal layer at the wound area (W). (b-d) Non-treated wound, 5 days post wounding showing disorganized re-epithelization (Ep) of the epidermal layer, dermal inflammatory cells infiltration (IF) along with proliferated granulation tissue, edema (E) and irregularly deposited collagen bundles (Co). (e and f) UDCA-treated wound 5 days post wounding showing subsiding of the inflammatory zone at the edges of the wound with well packed collagen fibers (Co) and advanced re-epithelization of the epidermis (Ep). **(B).** Van Gieson’s stained wound sections for type 1 collagen fibers 5 days post-incision revealing; dense and well aligned collagen deposition in non-treated wound (a and b) and in UDCA (c) treated wounds. (d) Quantitative analysis of area percent of type1 collagen fibers showing more significant (*P<0*.*05*) deposition of collagen fibers in UDCA- treated group than the corresponding untreated group at 5 days post-wounding. UDCA: ursodeoxycholic acid. * P<0.05, **P < 0.01: significantly different from untreated control.

Microscopic examination of skin wounds at zero-day post-wounding revealed destructed epidermal layer (Fig 3A). While 5 days post-wounding, the skin wound of the non-treated group showed re-epithelization of the epidermal layer which appeared disorganized with some thickened areas (Fig 3B). The keratin layer was either absent or decreased in thickness. The dermal layer under the wound area showed diffuse inflammatory cells infiltration along with proliferating granulation tissue, congestion and oedema (Fig 3C). Sometimes collagen bundles with slight disorganization were observed to be deposited in the vicinity (Fig 3D). On the other hand, the use of UDCA in the treated group improved the process of healing as shown by advanced re-epithelization and keratinization of the epidermis, as well as dermal regeneration represented by subsiding and retraction of the inflammatory zone at the edges of the wound (Fig 3E) with well-packed collagen fibers (Fig 3F) and increased number of hair follicles.

The morphometric analysis and scoring of the degree of re-epithelization, area percent of collagen fibers, and number of inflammatory infiltrates as well as the number of hair follicles in both untreated and UDCA-treated groups at 5 days post-wounding are presented in Table 1.

**Table 1:** The scoring of various morphological parameters of the skin wounds at 5 days post-wounding.

The analysed parameters Group	Score of re-epithelization	Score of collagen fibers deposition	Number of infiltrated cells with clear nuclei	Number of hair follicles
Non-treated group.	0.48± 0.25	0.48± 0.25	68.2± 1.76	4.4± 0.13
UDCA-treated group	2± 0.32**	2± 0.31**	27.6± 1.63**	6.4± 0.4**

Data were statistically compared by student *t-test*

* P<0.05

**P < 0.01: significantly different from untreated control.

Quantitative analysis of the area percent of type1 collagen fibers stained by Van Gieson’s stain revealed significant (*P<0*.*05*) more collagen fibers deposition in the UDCA-treated group than in the corresponding untreated group at 5 days post-wounding (Fig 3B).

**Fig 4:**
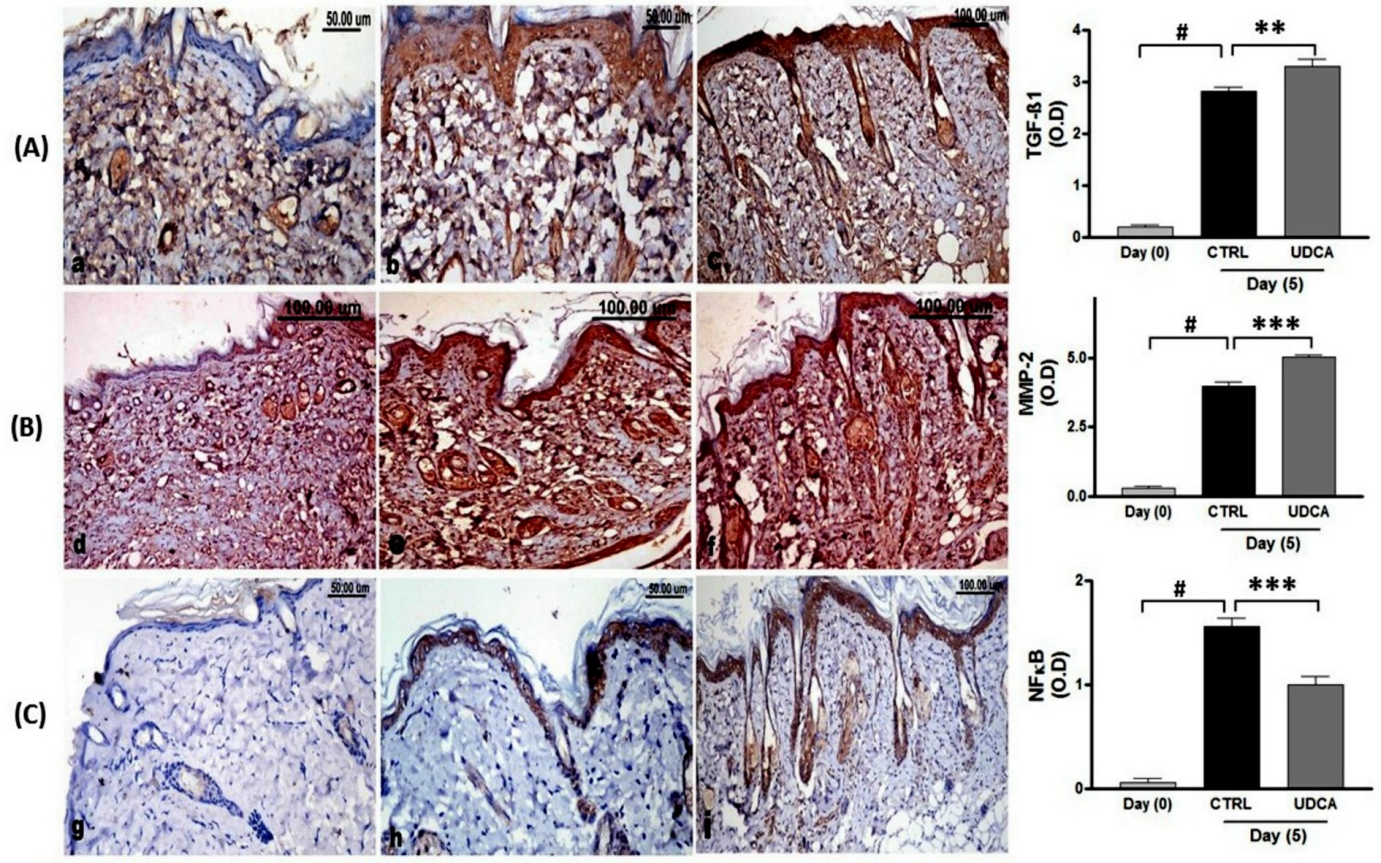
**(A)** TGF-β1 immune-stained wounded skin sections revealing; decreased expression in at zero-day(a), significant increased expression in non-treated (b) and in UDCA-treated (c) wounds 5 days post wounding. **(B)**MMP-2 immune-stained wounded skin sections’ revealing low immunopositivity at zero-day (a), significant increased immunopositivity in non-treated (b) and in UDCA-treated (c) wounds5 days post wounding. **(C)** NF*-*κB immune-stained wounded skin’ sections revealing; nil expression at zero-day(a), increased expression in the non-treated group(b) more than in the UDCA treated mice (c) at5 days post wounding. The quantitative image analysis, for TGF-β1, MMP-2 and NF*-*κB immune-staining expressed as optical densities (OD) across 5 different microscopic fields showing significant increased expression of TGF-β1 and MMP-2 with decreased the expression of NF-κB in UDCA-treated groups. *P<0.*05*, ***P < 0.*001*: significantly different from non-treated groups. UDCA: ursodeoxycholic acid.

Examination of the various immuno-stained wound sections revealed very low immunopositivity of TGF-β1 and MMP-2 with no expression of NF-κB at day zero. However, 5 days post-wounding, the expression of TGF-β1, MMP-2 and NF-κB was significantly increased compared with their expression at the zero day. The treatment with UDCA increased the expression of both MMP-2 and TGF-β1 and lowered the expression level of NF*-*κB (Fig 4).

### UDCA normalized the levels of oxidative stress markers and increased hydroxyproline content

**Fig 5:**
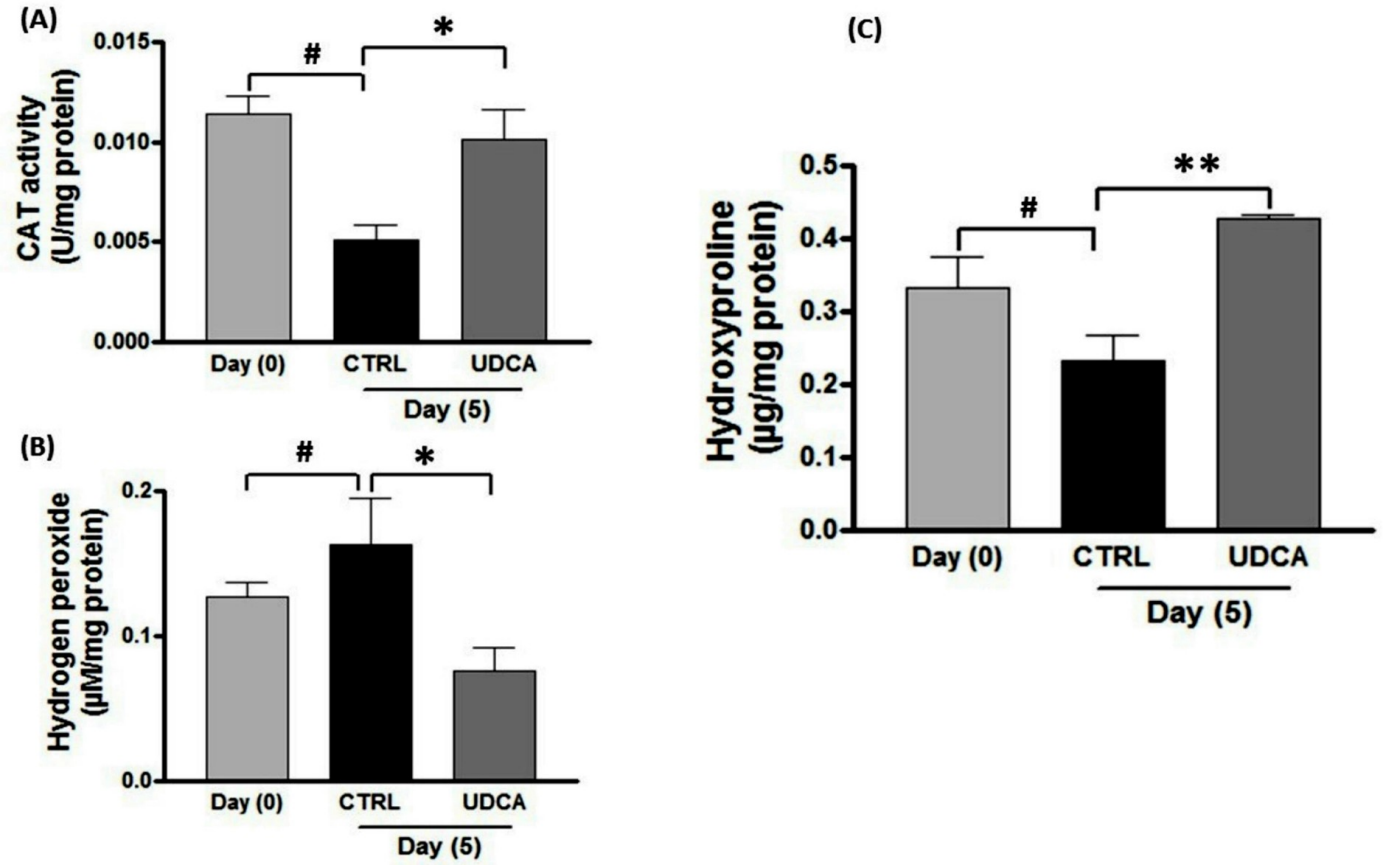
Effect of UDCA on biochemical analysis of (A) Catalase activity, (B) H_2_O_2_ and (C) hydroxyproline contents in skin wounds during different time points (zero, 5 day). Wound excision and application of treatment regimen (UDCA, 500μM cream) was carried out as mentioned in Fig 2. Error bars denote SEM for 6 experiments. *P<0.*05*, **P < 0.*01*: significantly different from untreated group. (#): significantly different from day (0) group. UDCA: ursodeoxycholic acid.

Compared with the non-treatment, application of UDCA for wounds significantly increased catalase activity by one-fold and significantly decreased hydrogen peroxide levels by 1.2 on day 5 post wounding (Fig 5A and 5B). On the other hand, estimation of hydroxyproline content (an index of collagen content and new collagen synthesis) revealed significant increase in the UDCA-treated group compared with the untreated controls at day 5 (Fig 5C).

### UDCA modified the energy consumption rate during wound healing

**Fig 6:**
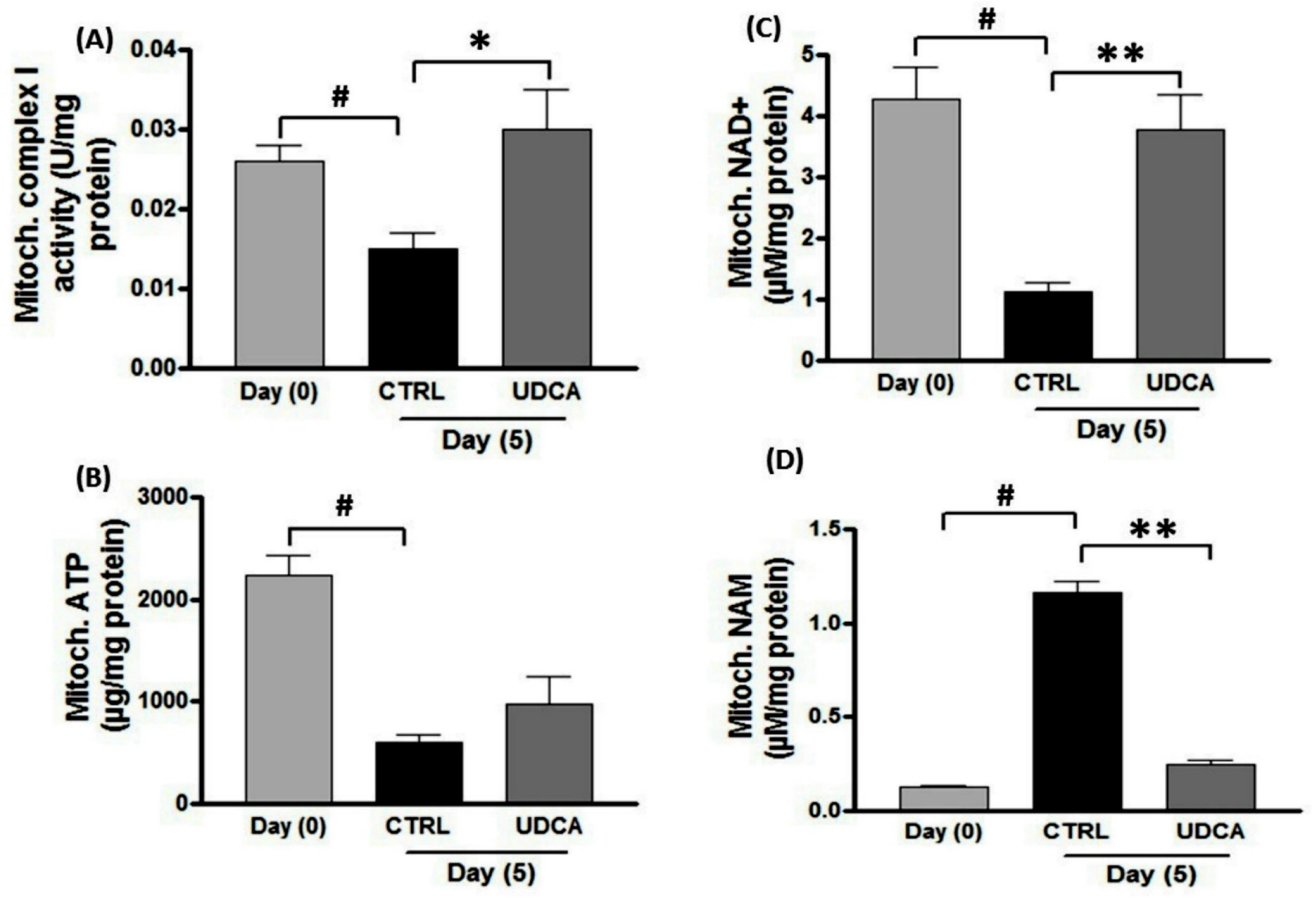
Effect of UDCA on mitochondrial functions. (A) Complex I activity, (B) ATP, NAD^+^ (C) and (D) NAM were determined in isolated mitochondria from collected samples among different time intervals (zero and fifth days). Wound excision and application of treatment (UDCA, 500μM cream) was carried out as mentioned in Fig 2. Error bars represent SEM (*n = 6)*. *P<0.*05*, **P < 0.*01*: significantly different from untreated group. (#): significantly different from day (0) group. ATP: adenosine triphosphate, NAD^+^: nicotinamide adenine dinucleotide, NAM: nicotinamide, UDCA: ursodeoxycholic acid.

As shown in Fig 6, skin injury showed a marked consumption in mitochondrial ATP and as well as complex I activity related to normal zero day. Moreover, treatment with UDCA showed further enhanced mitochondrial activity of complex I (Fig 6A). In parallel, mitochondrial ATP content showed one-fold increase in wounded skin after application of UDCA on the 5^th^ day post-wounding (Fig 6B). Interestingly, the cellular production of NAD and NAM showed reverse interchangeable levels on UDCA-treated wounds compared to the untreated control.

### Topical application of UDCA accelerated wound healing on chronic model of gamma irradiated wounds

**Fig 7:**
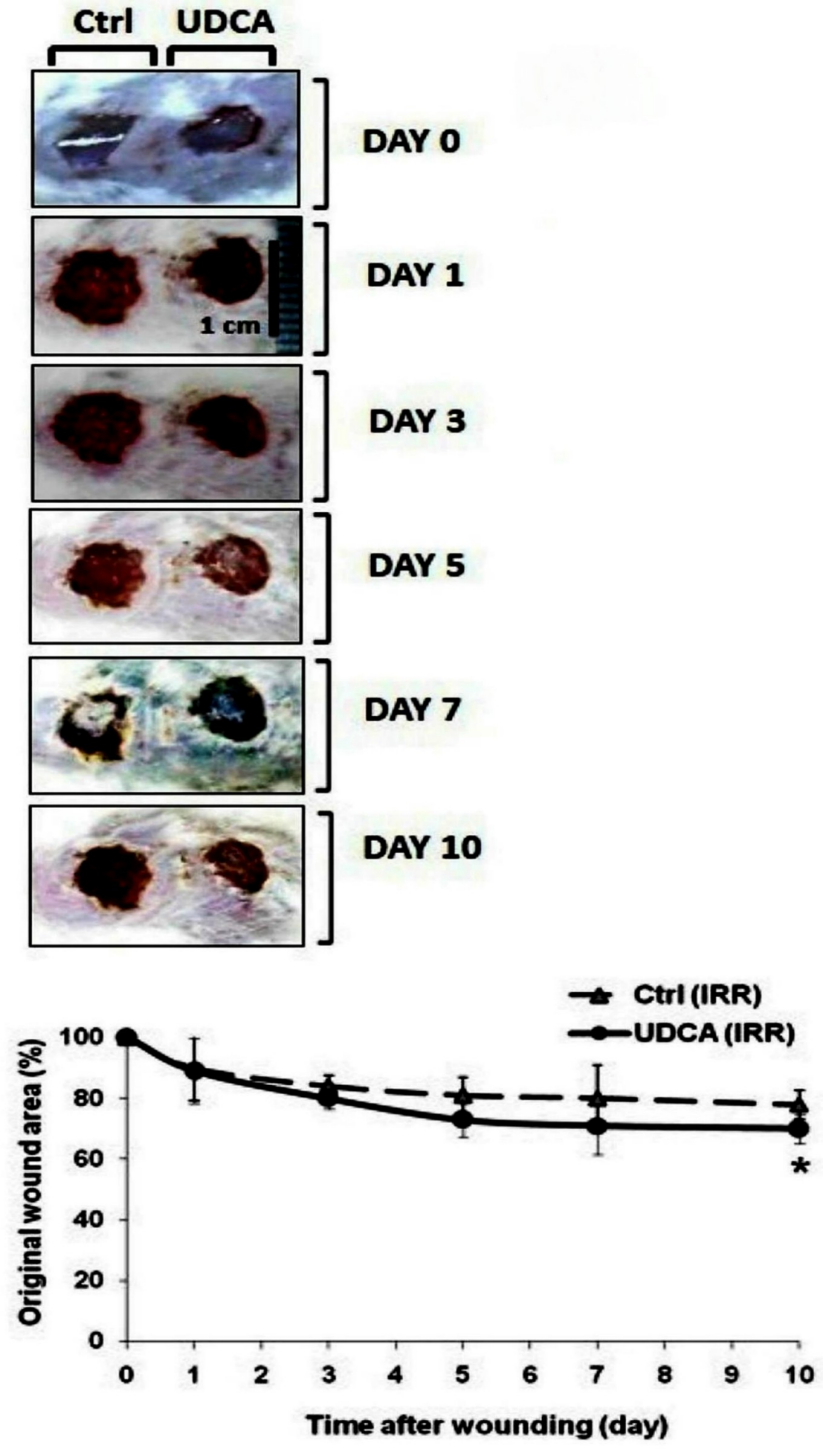
Effect of topical application of UDCA on chronic model of gamma irradiated wounds. Animals were pre-whole-body gamma irradiated (6Gy). Wound excision and application of treatment (UDCA, 500μM cream, starting 24h after gamma-irradiation) was carried out as mentioned in Fig 2. Photos of wounds were captured every day starting from day 0 (wounding). The line graph represents the mean of percent change relative to original wound size ± SE median (n = 6). *P<0.*05*: significantly different versus vehicle-treated (dashed line) wounds in the same time point. IRR: irradiated, UDCA: ursodeoxycholic acid.

It was observed that the normal healing of the excised wounds was delayed by exposing mice to gamma radiation (S1 Fig). UDCA did an acceleration of wound closure obviously starting from day five after wound creation and significantly showed 50% difference on the 10^th^ day as for untreated wounds (Fig 7).

**Fig 8:**
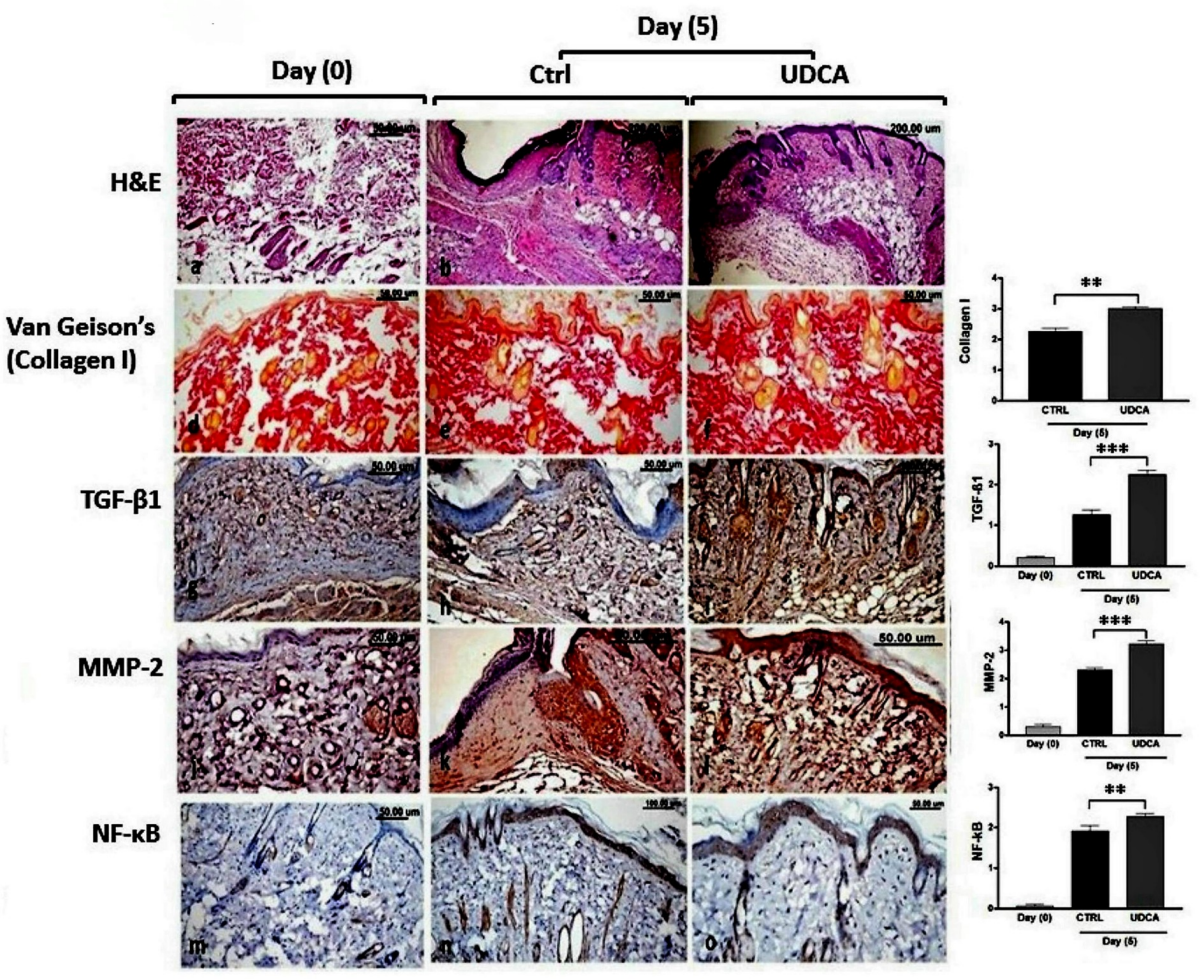
**(a-c)** H&E stained skin wounds sections of irradiated mice showing; (a) markedly destructed epidermal and dermal layers at zero-day post wounding. (b) disorganized and crinkle epidermal layer with numerous degenerated areas and scab and dermal inflammatory infiltrates and edema in non-treated wound at 5 days post wounding. (c) good, scarcely irregular re-epithelization and somewhat organized collagen fibers at 5 days post wounding in UDCA-treated wound. **(d-f)** Van Gieson’s stained sections showing; destructed and disorganized collagen fibers in non-treated wound (d and e) and its more deposition in UDCA-treated wounds (f). The quantitative analysis of area percent of type1 collagen fibers showing significant (*P<0*.*05*) more deposition of collagen fibers in UDCA- treated wounds. **(g-l)** Decreased expression of TGF-β1 and MMP-2 at day zero (g and j) and its increase at day 5 in non-treated wound (h and k) with more significant increase in both markers’ expression in UDCA-treated wounds (i and l). (m-o) Scares expression of NF-κB at zero day (m), and increased expression in both non-treated and UDCA-treated wounds 5 days post wounding.

As presented in Fig 8, microscopic examination of the irradiated mice at zero-day post-wounding revealed a markedly destructed epidermal layer with tissue disorganization (Fig 8A). At 5 days post-wounding, several histological alterations were observed as distorted, disorganized, crinkled and sometimes atrophied epidermal layer with a highly abnormal thickened keratin layer. The dermal layer appeared oedematous and widened with numerous degenerated areas (Fig 8B). Some hair follicles were atrophied and damaged. The skin of irradiated and UDCA-treated mice 5 days after wounding showed some aspects of improvement in wound healing with good, sometimes irregular re-epithelization and a very thin keratin layer, while the dermal layer showed organized collagen fibers (Fig 8C), sometimes with few focal inflammatory infiltrates.

Furthermore, the area percent of type1 collagen fibers stained by Van Gieson’s stain revealed significant (P<0.01) more collagen fibers deposition in the UDCA-treated group in gamma-irradiated animals (Fig 8D–8F). Assessment of immuno-stained wound sections from irradiated mice showed marked increase in TGF-β1 (Fig 8G–8I) and MMP-2 (Fig 8J–8L) expression levels upon application of UDCA compared with untreated wounds. On the other hand, UDCA did not exert a much significant change in NF-κB levels compared with UDCA-untreated injuries (Fig 8M–8O).

## Discussion

The common adverse unhealed skin injuries associated with many pathological conditions (e.g. diabetes and cancer) make the urge to explain the perspective signalling pathway in solving such clinical problems. Despite many investigations reporting the major mechanisms of cutaneous wound healing and the underlying complications associated with delayed healing, it is imperative to discover new agents that could be clinically efficacious for the reconstructing and remodelling of such unhealed wounds. Many biological and molecular events are involved among consecutive and overlapping phases of the healing process, namely, inflammation, proliferation and remodelling.

NF-κB and TGF-β1 are two master regulators of the transcription of inflammatory mediators that are released and triggered the initiation of fibroplasia and collagen deposition [2]. The treatment approach for delayed wound healing using UDCA is based on its biological function in modulating the activation of NF-κB [12]. In the current study, we precisely tested the effect of UDCA application on the propagation of pathways in a model of wound healing via TGF-β1/fibroplasia/collagen deposition. The somewhat interesting finding of accelerated wound closure and overall improvement in histopathological scores upon application of UDCA even after gamma radiation may be due to a combination of effects on various cellular events that are reporting for healing process, as discussed below.

Oxidative stress has been long accepted as a participant in delayed wound healing **[25].** Principally, stressful oxidative condition and overproduction of free radicals provoke the cascade of healing process. Other studies showed that agents that can stimulate chronic wound healing after the course of ionizing-irradiation may increase the probability of survival in experimental animals **[26].** In the current work, excised wounds were associated with unbalanced oxidative stress, modification of free radical release and upregulation of NF-κB, a master key of inflammatory mediators' expression; the factors that were corrected by topical application of UDCA. Many biological factors (e.g. inflammatory cytokines), oxidative stress, external stimuli (e.g. bacterial infections and gamma-radiation) or pathophysiological conditions (e.g ischemia) have been associated with activation of NF-κB supporting its role for elaboration of plenty signaling pathways that regulate the different biological functions **[27].** The translocation of NF-κB into the nucleus in turn regulates the expression of other pro-inflammatory cytokines, growth factors, and differentiation-regulating mediators, the signals mandatory for wound healing. However, such healing ability of UDCA to chronic irradiated model of induced wounds did not appeared to happen through NF-κB inhibition-dependent mechanism; nevertheless, it is concluded for the non-irradiated wound. On the other hand, the respiratory chain reaction is chiefly responsible for mitochondrial ROS production, particularly through complexes I and III **[27].** ROS production under physiologic conditions are decomposed by natural defence mechanisms principally achieved by mitochondrial and cytosolic superoxide dismutase, which neutralizes superoxide anion to hydrogen peroxide that scavenged by catalase or glutathione peroxidase at the expense of GSH **[28].** However, many pathological conditions may bring about an excessive ROS generation and impaired antioxidant defence system. These facts are reflected in the current study, where the antioxidant system was impaired during the wound healing process, as indicated by a reduction in catalase activity. In parallel, a pronounced increase in the cutaneous content of hydrogen oxide was also observed. These observations are in agreement with our previous findings **[3].** On the other hand, UDCA can neutralize such an oxidative imbalance. Additionally, previous studies such as Lapenna et al. **[9]** concluded similar results using different models of oxidative stress. Combined data analysis also revealed a further restoration in mitochondrial parameters for UDCA-treated wounds in terms of ATP and complex I activity, which is a normal compensatory response to the energy required for cellular migration and proliferation. Multiple studies have demonstrated the role of UDCA in mitochondrial biogenesis in different experimental models, such as parkinsonism [9] and hepatitis **[29].** These observations were in line with the present finding of increased fibroblast migration upon incubation with UDCA compared with the non-treatment, indicating an effect of UDCA related to energy restoration. Nicotinamide adenine dinucleotide (NAD) is a key cofactor essential for cell metabolism, biosynthesized by *de novo* pathway from tryptophan, and the continual consumption and degradation to nicotinamide (NAM) especially in pathological conditions stimulates the necessary intercellular NAD-salvage pathway **[30].** Here, UDCA by virtue of its energy restoration, revealed by elevated levels of ATP and complex I activity, increased the intracellular contents of NAD with lower degradation to its limiting end product, NAM as compared to untreated control.

In general, fibroplasia depicts the process of proliferation of fibroblast, formation of new collagen with other matrix proteins and fibroblastic migration into the wound fibrin clot, which eventually ends by granulation tissue formation, as soon response to injury insult **[31].** Five days later, migration of fibroblasts starts directly into the temporary wound clot matrix, where they lay down a matrix that is rich in collagen **[32].** Basically, mitochondrial biogenesis and intracellular localization are associated with different cell lines migration **[33].** One possible explanation was the AMP-activated protein kinase might support the increased energy demand during cell migration by regulation of mitochondrial redistribution and biogenesis **[34].** Of note, at high concentrations, UDCA significantly inhibits cell proliferation and promotes cellular death **[35].** While UDCA inhibition of NF-κB caused by reducing the nuclear activation of p65 **[36]** was noted as a potential growth-proliferation prevention in malignancy, UDCA may exert its action by down-regulating the inflammatory mediators. Hence, UDCA is ratified to have a potential curative application in diseases of inflammatory nature as well as cancers **[36, 37].**

Histopathologically, the use of UDCA markedly improved the healing process as shown by faster wound closure than that in the untreated groups. The degree of re-epithelization and area percent of collagen fibers were higher on using UDCA which can be attributed to TGF-β1 and gelatinase A (MMP-2) which showed significantly increased immuno-expression in our work. Gelatinases could be used as indicators for the progression of wound healing process. Gill and Parks **[38]** supported the importance of MMP in cell migration and re-epithelization during cutaneous wound healing in micro-dissected rat wound tissues. Additionally, TGF-β1 plays a major role in decreasing the proliferation of basal keratinocytes while enhancing the differentiation of supra-basal cells, thereby stimulating epidermal regeneration that is associated with cutaneous healing **[38, 39].** TGFs can initiate the fibroblastic conversion into myofibroblasts which are in charge of wound contraction and additional matrix proteins deposition **[40].** In the current work, the skin tensile strength of UDCA-treated and non-treated wounds was evaluated in terms of the amount and arrangement of collagen fibers, which showed steady organization in the UDCA-treated groups. Collagen is a major extracellular matrix (ECM) constituent for the maintenance of skin tensile strength and elasticity, and the increase in wound tensile strength that occurs during wound healing is usually associated with increased levels of collagen within the wound [39]. Many studies have greatened the role of increased production of collagen in wound healing **[41].** In the current study, Van Gieson’s staining of tissue sections from wound revealed that UCDA exerted positive effect on the synthesis of type1 collagen fibers. Overall, histological examination revealed that UCDA application resulted in more significant epithelialization and type1 collagen fiber content compared to the control group. Moreover, the contraction of the wound area exhibited a more rapid repair of the wound in UCDA treated group than the control group. Consequently, all together share to increase in tensile strength as a result of increase type1 collagen fibers which confer strength to tissue and increase the rate of epithelialization.

Moreover, the current study also indicated increased expression levels of fibrogenic (TGF-β1) to fibrolytic (MMP-2) during altered activity of NF-κB in both gamma-irradiated and non-irradiated wound models and hence increased collagen formation. A previous study examined the interplay between NF-κB pathway activation and excessive collagen deposition through NF-κB-independent signalling abnormalities that increased the relative expression of fibrogenic to fibrolytic mediators in specific dystrophic muscles in the mdx respiratory musculature *in vivo* model **[42].**

In the current study, we provided a model of radiation-impaired wound healing to elucidate the capability of locally applied UDCA in accelerating the healing process. Exposure to ionizing radiation is inevitable nowadays in wide range of medical diagnostic purposes, even though, it could be a hazard of occupational exposure. As pointed out in this study, repetitive radiation injury interrupts the organized events of wound healing, resulting in repetitive inflammatory responses and ongoing cellular regeneration. In irradiated tissue, fibroblasts have been shown to generate a disorganized deposition of collagen bundles. Significantly, UDCA accelerated wound contraction on either normal or impaired wound healing. Moreover, the present work showed also a merit of UDCA-local application on wounds. Based on provided observations, we believe that the utility of UDCA-carried dermal delivery system is an attracting medical approach among dermatologists in the future.

In general, UDCA increases expression of antioxidants that prevent toxic bile acids from causing DNA damage and cellular death **[43];** the phenomena which was revealed in our study on UDCA-modified oxidative stress in wounded skin. The anti-apoptotic effects of UDCA were initially observed in hepatocytes, not only due to an inhibition of ROS production but also by avoiding the release of pro-apoptotic factors from the mitochondria. The observations that was early elucidated by Rodríguez et al **[44]** on isolated rat enterocytes.

In conclusion, the overall data presented in the current work are in line with the investigated functions of UDCA in different apoptotic and inflammatory models. UDCA may accelerate wound contraction by correcting the oxidative imbalance, revealed a decrease in NF-κB expression, suggesting that even partial reduction in inflammatory mediators’ expression might has a favourable effect in enhancing the healing process. Also, UDCA enhanced collagen deposition and fibroblast migration explained by increased expression of TGF-β1 and MMP-2 as well as the reservation of energy consumption. Importantly, NF-κB expression was not much changed upon UDCA application in our case of delayed irradiated wound model. Even though, a pronounced acceleration in the healing process was observed.

## Supporting information

S1 Fig Dose effect of UDCA on wound contraction in (6Gy) gamma-irradiated and non-irradiated mice. IRR: irradiated, NR: non-irradiated, UDCA: ursodeoxycholic acid.
(TIF)
