# Supplementary figures and images for: Potential effects of ursodeoxycholic acid on accelerating cutaneous wound healing
Source: PLoS One. 2019 Dec 23;14(12):e0226748. doi: 10.1371/journal.pone.0226748 (PMC6927640; doi:10.1371/journal.pone.0226748)

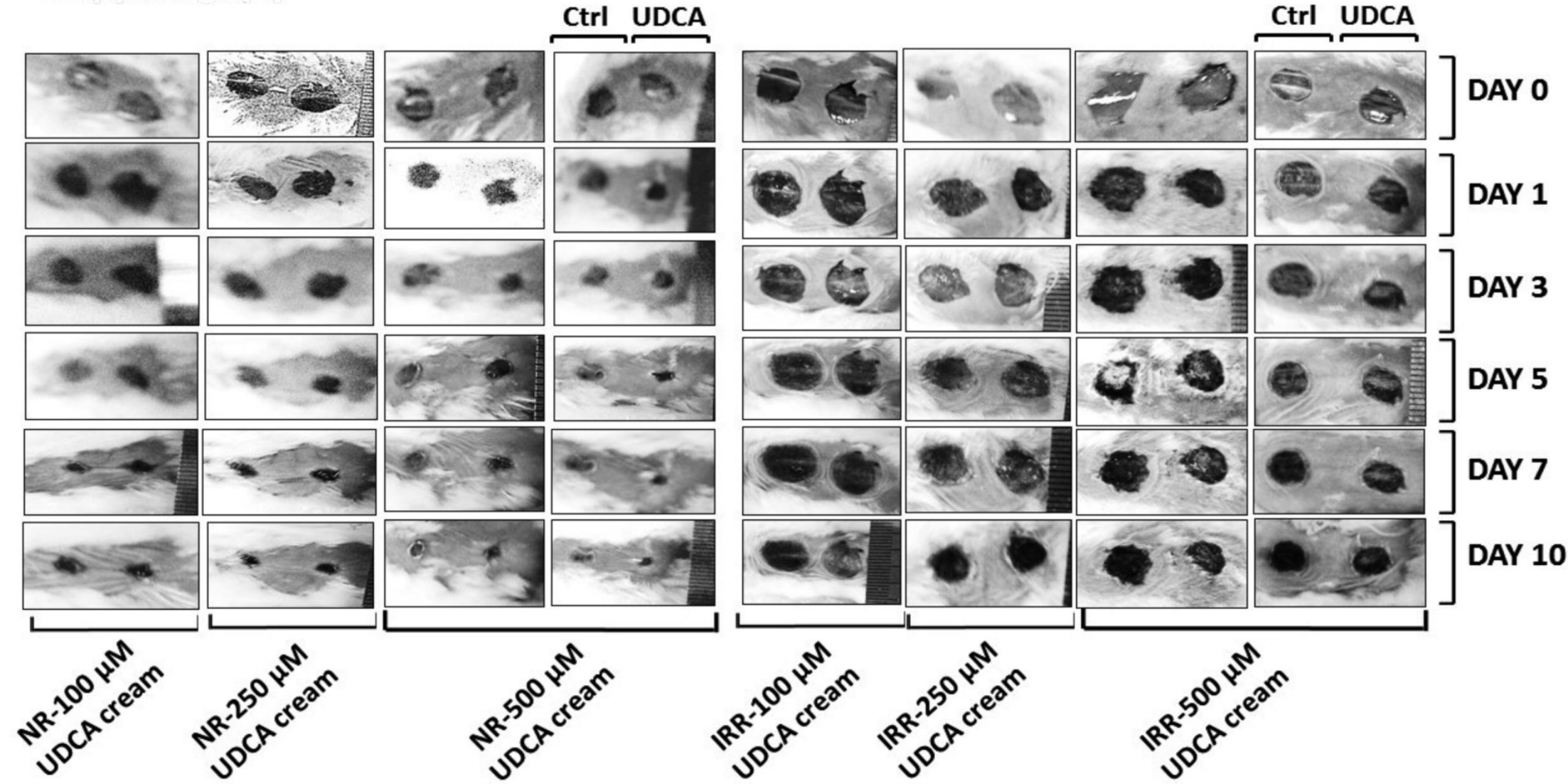

Supplement: S1 Fig — IRR: irradiated, NR: non-irradiated, UDCA: ursodeoxycholic acid. (TIF) [file pone.0226748.s001.TIF]
